# Supplementary material for: Negation detection in Dutch clinical texts: an evaluation of rule-based and machine learning methods
Source: BMC Bioinformatics. 2023 Jan 9;24:10. doi: 10.1186/s12859-022-05130-x (PMC9830789; doi:10.1186/s12859-022-05130-x)
Supplement: Supplementary file 1 — Additional file 1: A. description of datasets using text examples. S1. Comparison of different RobBERT/RoBERTa versions, using a batchsize of 32, 3 epochs, a gradient of $10^{-4}$10-4 and a maximum sequence length of 512. S2. Overview of RobBERT/RoBERTa results for different batch sizes and token windows. S3: Information on the corpora that were used for the Domain-Adapted-Pre-Training [file 12859_2022_5130_MOESM1_ESM.pdf]

Supplementary Material

A Text examples

We briefly describe the records used for our analysis, in order of least-to-most structured;

General Practitioner (GP) entries are clinical notes taken by the GP (a primary care physician) during a patient visit.

Specialist letters are longer-form records written by a medical specialist (e.g. a cardiologist), to report back to the GP after their patient has been referred to a hospital. Both of these record types come from the IPCI database (de Ridder et al. 2022), a large collection of medical records from Dutch GPs. The GP entries and (to a lesser extent) the specialist letters are not grammatically well-formed and often contain clinical shorthand.

Radiology reports are written by the radiologist to communicate findings and conclusions from diagnostic imaging to the physicians (specialists or GPs) that requested them.

Discharge letters are intended to inform GPs and other physicians on a hospital admission episode for a given patient. Both the Radiology reports and Discharge letters originate from the Erasmus Medical Center only, and are grammaticaly well-formed and easier to understand by others (especially the Discharge letters) compared to the GP entries and the specialist letters.

The following are representative examples of each record type in the Erasmus Medical Center Dutch Clinical Corpus (Afzal et al. 2014). An approximate English translation is printed underneath; the annotated medical terms are in **bold**.

A.1 GP Entries

al 6 d temp 39- 40 graden, **hoesten**, neusverkouden, eet wel OMA re , li een buisje, rustig, forse rhonchi diffuus mogelijk viraal, maar gezien de lange duur nu toch ab

already 6 d (*days*) temperature 39-40 degrees, **coughing**, runny nose, does eat Otitis Media Acuta ri (*right*), le (*left*) a tube, calm, considerable rhonchi diffuse possibly viral, but considering the long duration now after all ab (*antibiotics*)

A.2 Specialist letters

PED Sinds 3 jr is #Name# regelmatig **misselijk** en braakt hij frequent, gerelateerd aan **stress** en spanningen; Volledig bloedbeeld en bezinking: g.a.; HP negatief.
Coeliakie neg.

PED (*pediatrics*) Starting 3 yr (*years*) ago #Name# (redacted) is regularly **nauseous** and vomits frequently, related to **stress** and tension; Complete blood count and sedimentation; n.a. (no abnormalities); HP (*Helicobacter Pylori*) negative. **Celiac disease** neg. (*negative*)

A.3 Radiology reports

KLINISCHE INFORMATIE: Op 3 januari plotseling **hoofdpijn**.
Toch **intracraniale bloeding**?
VRAAGSTELLING: Toch **aneurysma**?
Er is multifocaal **spasme** in linker A. cerebri posterior en rechter A. cerebelli superior. pericallosa distaal is multifocaal **spasme**.
Differentiaal **diagnose** moet gesteld worden tussen **vasculitis** en **spasme** door subarachnoidale **bloeding**.
Een **aneurysma** is niet aangetoond.

*CLINICAL INFORMATION: On January 3rd suddenly **headache**. **Intracranial hemorrhage** after all? INDICATION: **Aneurysm** after all? There is multifocal **spasm** in left A. cerebri posterior en right A. cerebelli superior. pericallosa distal is multifocal **spasm**. Differential **diagnosis** should be made to distinguish **vasculitis** from **spasm** because of subarachnoidal **hemorrhage**. There is no evidence for an **aneurysm**.*

A.4 Discharge letters

De **diagnose** distorsie mediale band werd gesteld en patiente kreeg een gipskoker voor 4 weken, daarna fysiotherapie.
Daarna trad **zwelling** op.
De linker knie toont enige kapselzwelling maar geen **hydrops**.
Diagnose: Voornamelijk anterolaterale instabiliteit linker knie op basis van een oud voorste kruisbandletsel.

*The **diagnosis** sprained medial ligament was made and patient got a cast for 4 weeks, followed by physical therapy. Afterwards **swelling** occurred. The left knee is showing some capsule-swelling but no **hydrops**. **Diagnosis**: Predominantly anterolateral instability left knee on the basis of old anterior cruciate ligament injury.*

B RobBERT tables

We see no consistent differences between the different RobBERT versions in this benchmark (Table S1). From Table S2 we conclude that using an almost ten times smaller maximum sequence length of 32 tokens gives comparable performance for the RobBERT models. Furthermore we see a performance comparable to the rule-based approach if we train only the final dense layer of the domain-specific MedRoBERTa.nl model. RobBERT does not benefit from the full 512-token size context window and lowering the context-window to 32 tokens only slightly reduces the accuracy[1] while drastically decreasing the computational requirement. Domain-adapted pre-training leads to a slight improvement.

Letter category	Prediction method	Precision	Recall	F1
Discharge letters	RobBERT	0.953	0.974	0.963
Discharge letters	RobBERT DAPT	0.964	0.984	0.974
Discharge letters	MedRoBERTa.nl	0.973	0.966	0.970
General Practitioner entries	RobBERT	0.950	0.912	0.931
General Practitioner entries	RobBERT DAPT	0.941	0.923	0.932
General Practitioner entries	MedRoBERTa.nl	0.943	0.923	0.933
Radiology reports	RobBERT	0.960	0.963	0.961
Radiology reports	RobBERT DAPT	0.956	0.960	0.958
Radiology reports	MedRoBERTa.nl	0.962	0.951	0.957
Specialist letters	RobBERT	0.934	0.890	0.911
Specialist letters	RobBERT DAPT	0.945	0.905	0.924
Specialist letters	MedRoBERTa.nl	0.936	0.915	0.925

Tab. S1: Comparison of different RobBERT/RoBERTa versions, using a batchsize of 32, 3 epochs, a gradient of 10^{-4} and a maximum sequence length of 512.

Prediction method	Precision	Recall	F1
RobBERT, 3 epochs, batch size 64, 512 tokens	0.94	0.90	0.92
RobBERT, batch size 32, 512 tokens	0.95	0.94	0.95
RobBERT DAPT, batch size 64, 512 tokens	0.94	0.93	0.94
RobBERT DAPT, batch size 32, 512 tokens	0.95	0.95	0.95
MedRoBERTa.nl, batch size 64, 512 tokens	0.95	0.92	0.94
MedRoBERTa.nl, batch size 32, 512 tokens	0.96	0.94	0.95
RobBERT, 3 epochs, batch size 32, 32 tokens	0.95	0.93	0.94
RobBERT DAPT, batch size 32, 32 tokens	0.95	0.95	0.95
MedRoBERTa.nl, batch size 32, 32 tokens	0.96	0.94	0.95
RobBERT simple-fit, 10 epochs, batch size 128, 512 tokens	0.89	0.65	0.75
RobBERT DAPT simple-fit, batch size 128, 512 tokens	0.90	0.75	0.82
MedRoBERTa.nl simple-fit, batch size 128, 512 tokens	0.94	0.81	0.87

Tab. S2: Overview of RobBERT/RoBERTa results for different batch sizes and token windows.

[1] when considered over the entities that could be classified

C DAPT text characteristics

Tab. S3: Text characteristics for domain adapted pre-training

characteristics	# of words	# of unique words	# of sentences with minimally 3 tokens
Huisarts & Wetenschap (1957-2019)	22.839,529	510,417	1.176,662
NHG directives/standards	17.052,394	257,030	921,336
FMS directives	41.254,869	325,357	2.188,601
Medical Wikipedia	4.928,938	213,565	285,617

Tab. S4: Information on the corpora that were used for the Domain-Adapted-Pre-Training

Bibliography

Afzal, Zubair et al. (2014). "ContextD: an algorithm to identify contextual properties of medical terms in a Dutch clinical corpus". In: *BMC bioinformatics* 15.1, pp. 1–12.

De Ridder, Maria et al. (2022). "Data Resource Profile: the Integrated Primary Care Information (IPCI) database, The Netherlands". In: *International Journal of Epidemiology*.
